# Comparing machine learning methods for predicting land development intensity

**DOI:** 10.1371/journal.pone.0282476

**Published:** 2023-04-05

**Authors:** Guanhai Gu, Bin Wu, Wenzhu Zhang, Rucheng Lu, Xiaoling Feng, Wenhui Liao, Caiping Pang, Shengquan Lu

**Affiliations:** School of Natural Resources and Surveying and Mapping, Nanning Normal University, Nanjing, China; Northeastern University (Shenyang China), CHINA

## Abstract

Land development intensity is a comprehensive indicator to measure the degree of saving and intensive land construction and economic production activities. It is also the result of the joint action of natural, social, economic, and ecological elements in land development and utilization. Scientific prediction of land development intensity has particular reference significance for future regional development planning and the formulation of reasonable land use policies. Based on the inter-provincial land development intensity and its influencing factors in China, this study applied four algorithms, XGBoost, random forest model, support vector machine, and decision tree, to simulate and predict the land development intensity, and then compared the prediction accuracy of the four algorithms, and also carried out hyperparameter adjustment and prediction accuracy verification. The results show that the model with the best prediction performance among the four algorithms is XGBoost, and its R^2^ and MSE between predicted and valid values are 95.66% and 0.16, respectively, which are higher than the other three models. During the training process, the learning curve of the XGBoost model exhibited low fluctuation and fast fitting. Hyperparameter tuning is crucial to exploit the model’s potential. The XGBoost model has the best prediction performance with the best hyperparameter combination of max_depth:19, learning_rate: 0.47, and n_estimatiors:84. This study provides some reference significance for the simulation of land development and utilization dynamics.

## 1. Introduction

Land development intensity is a comprehensive spatial mapping of the degree of intensive use of construction land, population carrying capacity, and human production activity, reflecting the organic unity of regional production, life, and ecological relations. Into the twentieth century, the urbanization of Chinese cities has made significant progress, and the scale of towns has been expanding. It also brings problems such as border disorder expansion, land use conflict, and inefficient land use [1–3]. However, pursuing high-quality regional development under the premise of scarce land resources and exploring good land development intensity and spatial layout are inevitable choices for healthy and sustainable socio-economic development and essential directions for future urban land use in China [4,5]. The report of the 19th National Congress proposes to set three control lines—ecological protection red line, permanent bare farmland, and urban development boundary, of which the urban development boundary naturally becomes the "ceiling" for the disorderly expansion of urban land scale [6]. Therefore, reasonably increasing the intensity of land development, tapping the urban stock of land, and remediating low-utility areas are the ways of regional green development [7,8]. Given this, to build a development pattern in the new era, promote high-quality development and achieve organic unity of social, economic, and ecological benefits of land use, the use of scientific forecasting means for land development intensity is of contemporary significance for the development of territorial spatial planning to depict the blueprint of urban development. It will help promote the overall orderly operation of the city and optimize the future pattern and utilization structure of land space development.

From the literature related to land use simulation and prediction, research methods have mainly used meta-automata models [9], system dynamics models [10], CLUE-S models [11], artificial neural network models [12], Markov chain models [13], etc., while integrated models have also been applied to improve the accuracy and reliability of prediction [14–16]. From the research object, the simulation prediction of urban construction land expansion gradually shifted from a single urban construction land expansion to a comprehensive simulation of multiple land types. With the continuous development of machine learning and deep learning, land use simulation and prediction models have been further improved, and many algorithms have been widely used for land change prediction, land cover classification, and target detection [17,18]. However, the spatial and temporal evolution of land development intensity is complex and systematic, and a combination of natural resource conditions, socio-economic conditions, and policies and institutions pose particular challenges to its prediction [19,20]. Following the introduction of machine learning algorithms for the prediction process of this complex land use dynamic simulation, it is more flexible compared with other traditional statistical models, mainly in terms of solid learning ability, a more efficient processing algorithm, generally does not need to consider problems such as multivariate covariance, and is friendly to nonlinear data processing. In addition, machine learning algorithms have the advantages of higher prediction accuracy, faster convergence, and fewer adjustment parameters while effectively avoiding problems such as overfitting and underfitting, and they have been widely used in many fields [21–24]. Therefore, it has obvious advantages for improving the simulation performance of systematic and complex spatio-temporal dynamic prediction of land development intensity.

**Fig 1:**
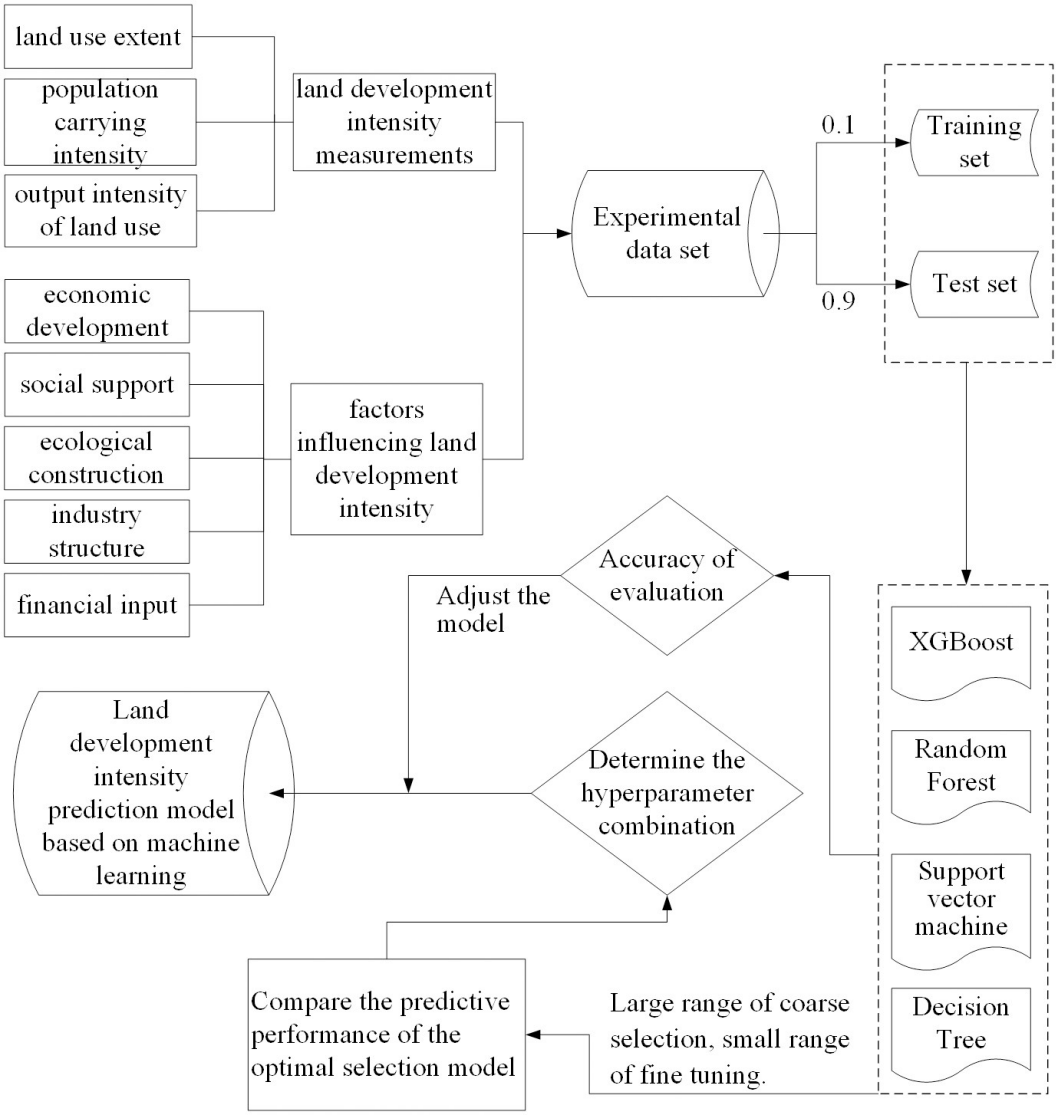
Research theoretical framework.

Overall, the existing research on dynamic simulation of land development utilization primarily employs traditional approaches such as Cellular Automata, the CLUE-S model, and the Markov Chain model. The application of machine learning algorithms for prediction and simulation is limited. Nevertheless, as a complex system, land development intensity reflects the quality and efficiency of urban land use, shaped by multiple factors. Traditional prediction methods fail to provide a precise explanation of the variables and simulation of land development intensity. However, machine learning prediction based on big data leverages sophisticated algorithms and techniques, which significantly enhances the fitting accuracy of the data and produces more accurate predictions and simulations. For this reason, based on the above analysis, machine learning has the advantages of high learning ability, high accuracy, and a more intelligent process in predictive Simulation. Based on the theoretical framework of "data-experiment-model" (Fig 1), four machine learning algorithms, namely XGBOOST, random forest model, support vector machine, and decision tree, are used to train and test the accuracy of the sample data set of land development intensity in 31 provinces in China. The best model is finally compared and selected for land development intensity prediction, and the combination of hyperparameters and prediction results are validated based on the chosen algorithm. This research endeavors to showcase the efficacy of machine learning in predicting and simulating land development intensity, offering a reference for the field. The goal is to support the future development of the region and policy-making for urban land use planning, promoting regional economic growth, environmental sustainability and sustainable land utilization, and further advancing the studies related to land use change prediction and urban development.

## 2. Research method and research area

### 2.1 Study area

The study area of this paper includes 31 provincial administrative regions in mainland China, excluding Hong Kong, Macao, and Taiwan for the time being. Based on the mainland’s geographical location and natural environment, they are divided into seven regions: northeast, north, central, east, south, southwest, and northwest [25] (Table 1).

**Table 1:** Seven geographical regions of China.

Area	Provinces
Northeast	Liaoning, Jilin and Heilongjiang provinces
North China	Beijing, Tianjin, Hebei, Shanxi, and Inner Mongolia Autonomous Region
Central China	Hunan, Hubei and Henan provinces
East China	Fujian, Jiangxi, Zhejiang, Shanghai, Anhui, Jiangsu, Shandong
South China	Guangxi Zhuang Autonomous Region, Guangdong Province, and Hainan Province
Southwest China	Sichuan, Guizhou, Yunnan, Tibet Autonomous Region, and Chongqing
Northwest China	Shaanxi, Gansu, Qinghai, Ningxia Hui Autonomous Region, and Xinjiang Uygur Autonomous Region

### 2.2. Data source

The comprehensive reflection of land development intensity indicates the extent to which land is utilized in a region and its capacity to meet social, economic, and ecological needs. It is an index that encompasses multiple interrelated factors, which can be broadly classified into three categories: social and economic, ecological, and public service facilities [26–28]. On the social and economic front, economic growth plays a crucial role in urban development. A rapid increase in social and economic conditions leads to greater demand for land and population growth, causing an increase in the demand for city resources and infrastructure. Additionally, the development of social investment and industrial structures related to land use affects the land utilization structure. On the environmental level, the relationship between the natural environment and land development intensity is interdependent. The environment provides opportunities for land development, but also restricts it to some extent. At the same time, land development activities can alter local ecosystems and the environment. A good urban natural environment is crucial in attracting investment and promoting economic development, which in turn impacts land development intensity. The availability and quality of public service facilities directly impact land utilization and development in an area. Public facilities, which are essential to regional infrastructure, provide basic services to citizens. However, their limited ability to support increased population and economic activities may slow down land development. Hence, the relationship between public facilities and land development is complex, and the level of infrastructure may either promote or limit land development intensity.

We have chosen 16 indicators in the areas of economic growth, ecological environment, and public service facilities, to measure the factors influencing land development intensity. For measuring regional population distribution, industrial structure, and economic development, we have selected indicators such as per capita GDP (X1), the proportion of secondary and tertiary industries (X2), total retail sales of consumer goods (X3), population (X4), international tourism revenue (X5), per capita grain output (X6), and so on. To measure the region’s investment and public facility construction capability, we have selected indicators such as new construction land (X7), completed fixed asset investment in public utility facility construction (X8), general budget revenue (X9), per capita road area (X10), and total water supply (X11). To measure the level of regional infrastructure and public service construction, we have selected indicators such as green coverage area (X12), sewage treatment rate (X13), number of elementary and secondary school students (X14), number of public libraries (X15), and number of health institutions (X16).

2002–2020 socio-economic development level data and ecological environment category data are obtained from the China Statistical Yearbook (http://www.stats.gov.cn/tjsj./ndsj/) and the China Environmental Statistical Yearbook (https://www.mee.gov.cn/hjzl/sthjzk/sthjtjnb/), respectively. In addition, all the data of the article has been collated and shared on the following websites. The experimental data set, "Data set of provincial land development intensity and its influencing factors in China.xlsx," are available from figshare at: https://doi.org/10.6084/m9.figshare.21875622.v1.

### 2.3. Research method

(1) Land development intensity measurement

The intensity of land development (IOLD) is a comprehensive index that reflects the degree of construction and utilization of construction land in a particular area, the carrying level of population, and social and economic elements [29,30]. The formula is as follows:

IOLD=αCLUA+βPCC+λOIL (1)

Where *IOLD* represents land development intensity; *CLUA* represents the construction area of the unit and uses the ratio of the regional construction area to the total size of the site; *PCC* represents the ability to carry the population; *OIL* represents the intensity of land use, and is represented by the ratio of secondary and tertiary industrial values to the area of construction land; *α*, *β*, and *λ* respectively indicate the weight of the unit’s construction land, population bearing capacity, and the weight of land use intensity, and they are standardized through CLUA, PCC, and OIL, and then obtained by using entropy values. *α*, *β*, and *λ* are 0.4, 0.3, and 0.3, respectively.

(2) XGBOOST algorithm

XGBOOST is an advanced machine-learning algorithm. XGBOOST (Extreme Gradient Boosting) is an extreme gradient improvement tree. It combines multiple weak learning devices (decision trees) to iterate to generate a robust learning device. For the prediction of land development intensity that is commonly used by multi-factor, it can obtain a better return classification or simulation prediction. Compared with the general decision-making tree model, XGBOOST has improved the training effect by enhancing the study rate and the characteristics of the selection area, effectively preventing the risk of overfitting [31]. The formula is as follows:

$$J=\sum_{i=0}^{n}L(y_{i},{\bar{y}}_{i})+\sum_{k=0}^{k}\Omega (f_{k})$$ (2)

$$\Omega (f_{k})=\gamma T+\frac{1}{2}\mu \sum_{j=0}^{T}w_{j}{}^{2}$$ (3)

$$J^{m}=\sum_{i=0}^{n}L(y_{i},{\bar{y}}_{i}{}^{\left(m\right)})+\sum_{k=1}^{m}\;\Omega (f_{m}(x_{i}))=\sum_{i=0}^{n}L(y_{i},{\bar{y}}_{i}{}^{\left(m-1\right)}+f_{m}(x_{i}))+\sum_{k=1}^{m}\Omega (f_{m}(x_{i}))$$ (4)

Where *n* is the number of training samples, Ω(*f*_*k*_) is a regularized function, *T* is the number of leaf nodes *γ*, and *μ* is a defined hyperparameter in XGBOOST. *w* is the weight (the prediction value in the terminal node).

(3) Random forest algorithm

The random forest is an extended decision tree algorithm that combines the decision tree, but each is trained independently. In the case of multiple predicted factors, the inspection variable uncertainty changes in land development intensity. In addition, the random forest captures the potential interaction between the intensity of land development and influencing factors by biological data. The training process is generally a concentration of training data, drawing a guidance sample as a random set, and then each tree will use it. The random set of predicted variables grows as much as possible without trimming and then repeats the second step until the number of trees increases. Finally, the average predicted land development intensity is summarized [32].

(4) Support vector machine

Support vector machines are generally referred to as SVM, based on the principles of statistical learning theory, and are used to solve problems such as abnormal detection, clustering, turning guidance learning, regression, and classification. Establish a linear learning machine directly in high-dimensional characteristic space [33]. The formula is as follows:

$$y=f(x)=\sum_{i=1}^{m}w_{i}\varphi (x)=w\varphi (x)$$ (5)

$$y=f(x)=\left[\sum_{i=1}^{n}w_{i}K(x_{i},x)\right]-b$$ (6)

Where *K* is the core function; *w*_*i*_ is the model parameter; *b* is the constant; n is the number of training data; *x*_*i*_ is the input data set; *x* training network data vector.

(5) Decision Tree

The decision tree is a method that approaches the value of discrete functions, a typical classification method. As a prediction model, it can reflect a mapping relationship between the predictive value and the variable. The decision-making tree is a data classification process through a series of rules. Each tree consists of three parts, namely the root nodes, internal nodes, and internal nodes. Furthermore, leave the node. Each internal node represents a test of an attribute. Each branch from the root node to the leaf node represents the test output, and each leaf node represents a sample classification [34,35].

## 3. Experimental data processing and model selection

### 3.1. Data overview

**Fig 2:**
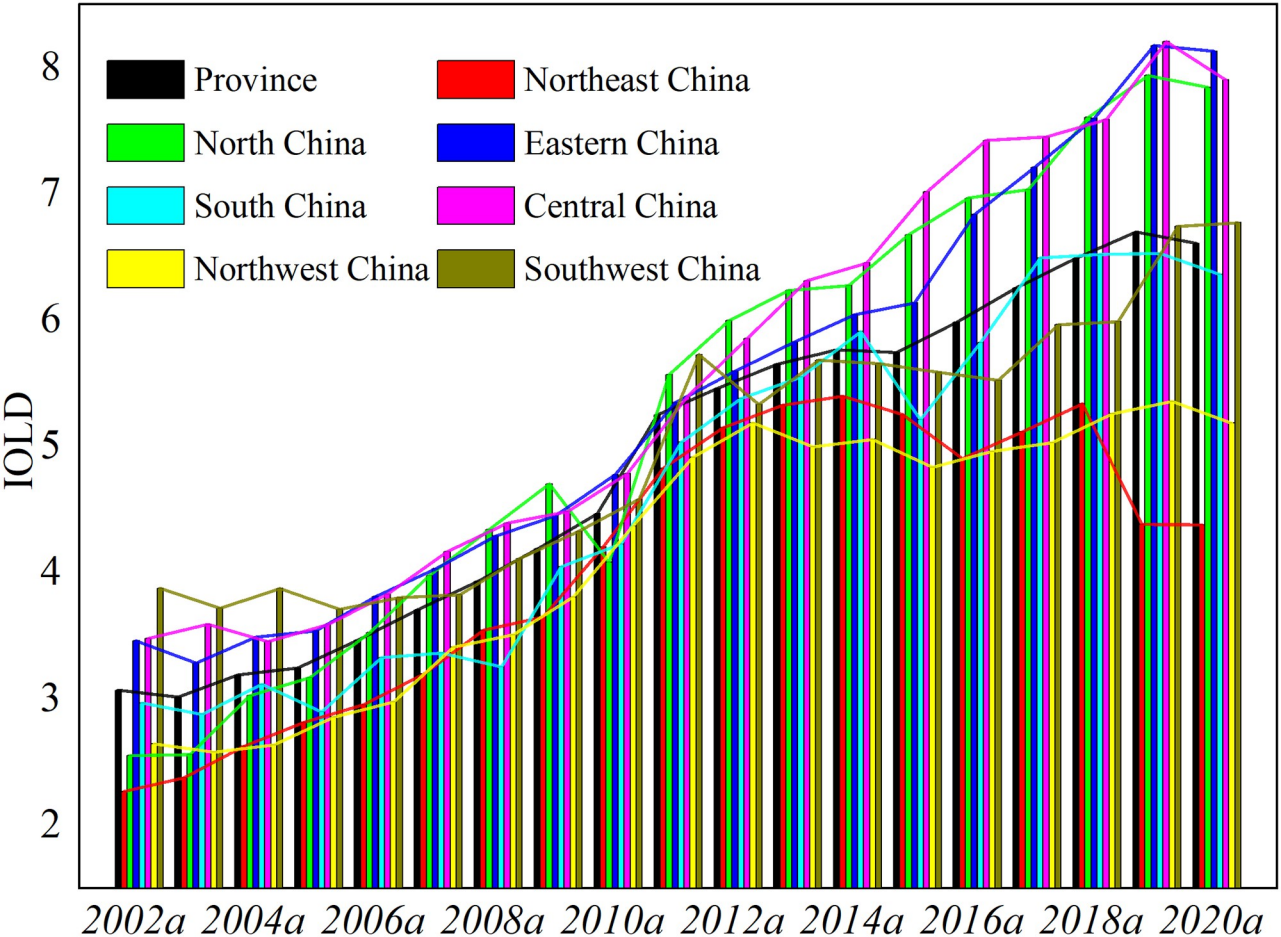
Mean change curve of land development intensity from 2002 to 2020.

Eq (1) was applied to measure the land development intensity of Chinese provinces from 2002 to 2020 as the experimental data for model construction, and the results were plotted as change curves (Fig 2). It can be seen that the average land development intensity of Chinese provinces during the study period is on an upward trend, with the moderate intensity increasing from 3.07 in 2002 to 6.61 in 2020a, with a steadily increasing growth rate from 2002 to 2011a in terms of the various stages of change. There is a significant decrease in the growth rate from 2012-2015a compared to the previous period, but the development intensity is still increasing; the growth rate of development intensity turns up from 2016-2019a. According to the regional division, land development intensity is higher in Central China, East China, and North China, followed by Southwest China, South China, and Northwest China, and the lowest in Northeast China.

### 3.2. Data processing

#### 3.2.1. Data test set

To construct a model that predicts land development intensity that is systematically influenced by multiple factors, we first set up an original experimental data set containing sample data on land development intensity and potential variables (factors influencing land development intensity) associated with the prediction target. Before training the model using the dataset, the machine learning algorithm analyzes all variables to filter out those that have little impact on the intensity of land development and those whose features are not obvious enough to be identified by the algorithm. For some elements, the model is used to select the most influential features with a considerable number of components in the aggregation process, such as type, distribution, size, and content, but some features may be too complex to be filtered in the database. In addition, when selecting a dataset, not all components can be considered. Otherwise, the results may be invalid [36]. Since the unit of study is at the regional scale, natural factors such as elevation, slope, climate, and other elements are challenging to express their characteristics with consistent data at a large scale, so biological factors are not considered in the potential variable dataset.

After the relevant literature analysis and the preliminary filtering of machine learning algorithms, we build a data set of land development intensity forecast, including the land development of 31 local administrative units in China from 2002–2020 Power and 16 variables. Then, the main component of the influencing factors of the land development intensity, using the principal component analysis for information concentration research and obtaining a set of data representation of the main component table (Table 2). Among them, KMO is more significant than 0.87, which indicates that the data can be used for the principal component analysis research and passed Bartlett spherical test. The absolute difference is explained to 89.92%, indicating that the indicator can better explain the intensity of land development.

**Table 2:** Main components of land development intensity factors.

KMO value	Bartlett’s sphericity test			Total variance explained
	Approximate cardinality	df	p	
0.87	12373.44	120	0.000***	89.92%

note:

***, **, and*represent the significant level of 1%, 5%, and 10%, respectively.

#### 3.2.2. Data set preparation

In the data collection process, the attribute characteristics of the sample data are processed. Since the prediction target is land development intensity, "land development intensity" is placed on the leftmost side of the data set as the "label" of the training example, and " Province" and "time" can be placed after the "label" as "group label," and the variables are placed after "label" as "feature." The model makes regression predictions based on the set data attributes of land development intensity and its influencing variables.

After the program starts, the algorithm automatically splits the data into training and testing subsets randomly based on a 0.9:0.1 ratio, and the randomness effectively avoids human interference, a process called data snooping bias. Then, the K-fold cross-validation method divides the training set into multiple subsets to optimize the training process by factorization, reduction, sampling, node splitting, etc. To filter whether there are non-significant variable features, adjust the hyperparameters, and then build a perfect simulation prediction model; the test set is an accuracy test of the model that has completed the training set, and the predicted value is compared with the actual value to evaluate the prediction accuracy of the built model. The test set is an accuracy check of the model that has completed the training set, and the model’s prediction accuracy is evaluated by comparing the predicted value with the actual value.

### 3.3. Model selection

After the above-mentioned experimental dataset settings and pre-processing work, the Python program is used to write the four algorithms: XGBOOST, Random Forest, SVM, and Decision Tree. The 90% land development intensity samples that are randomly divided are used as training data sets, and 10% test set import models. Finally, the prediction performance of the four models is compared to select the best algorithm for land development intensity prediction.

**Fig 3:**
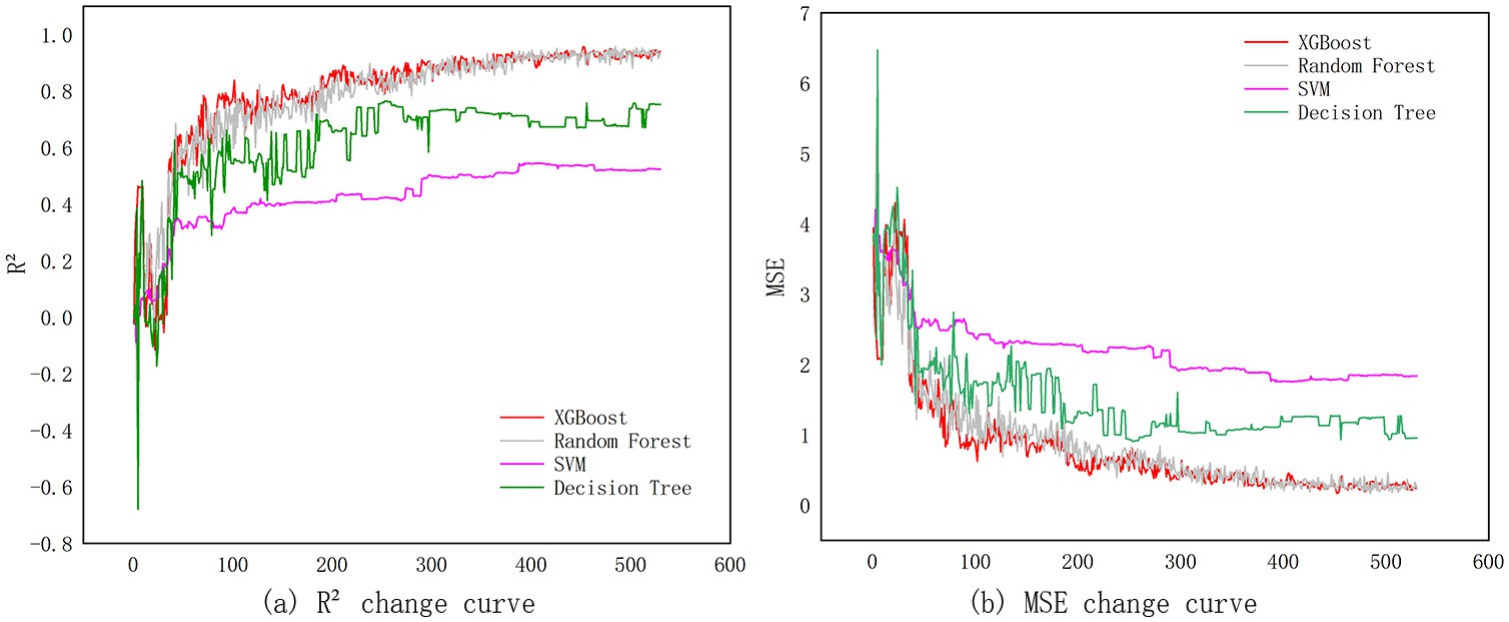
The four-model training learning curve.

First of all, from the change in the learning curve of the four models (Fig 3) and the model parameter (Table 3), the MSE (the predicted value of the square error, the difference between the prediction value and the actual value of the true value) and the R^2^ (measure the deviation of a set of data) are used to evaluate the performance of the model. If the smaller MSE, the larger the R^2^, the better the model can be explained. From the perspective of changes in the MSE curve of each model: SVM> D.T.> R.F.> XGBOOST; the size of R^2^ is: XGBOOST> R.F.> D.T.> S.V. The results showed that the predictive effect of the XGBOOST algorithm and the random forest model on land development intensity is better than that of decision trees and support vector machines. In addition, the XGBOOST algorithm is closer to the overall fluctuation of the random forest model from the model learning curve. However, from a small comparison, it is found that in the iteration range of 0–300, the advantages of the average error and data deviation of the XGBOOST algorithm are more significant, and the parameters of the two models are not much different. Therefore, on the whole, XGBOOST’s MSE is smaller, R^2^ is more significant, and the learning curve fluctuations are more stable. It is initially determined that the XGBOOST model predicts the best performance.

**Table 3:** Parameter comparison of the maximum prediction.

Models	XGBOOST	Random Forest	Support vector machines	Decision Trees
MSE	0.1622	0.1654	1.7555	0.9075
R^2^	95.66%	94.72%	54.43%	76.44%

**Fig 4:**
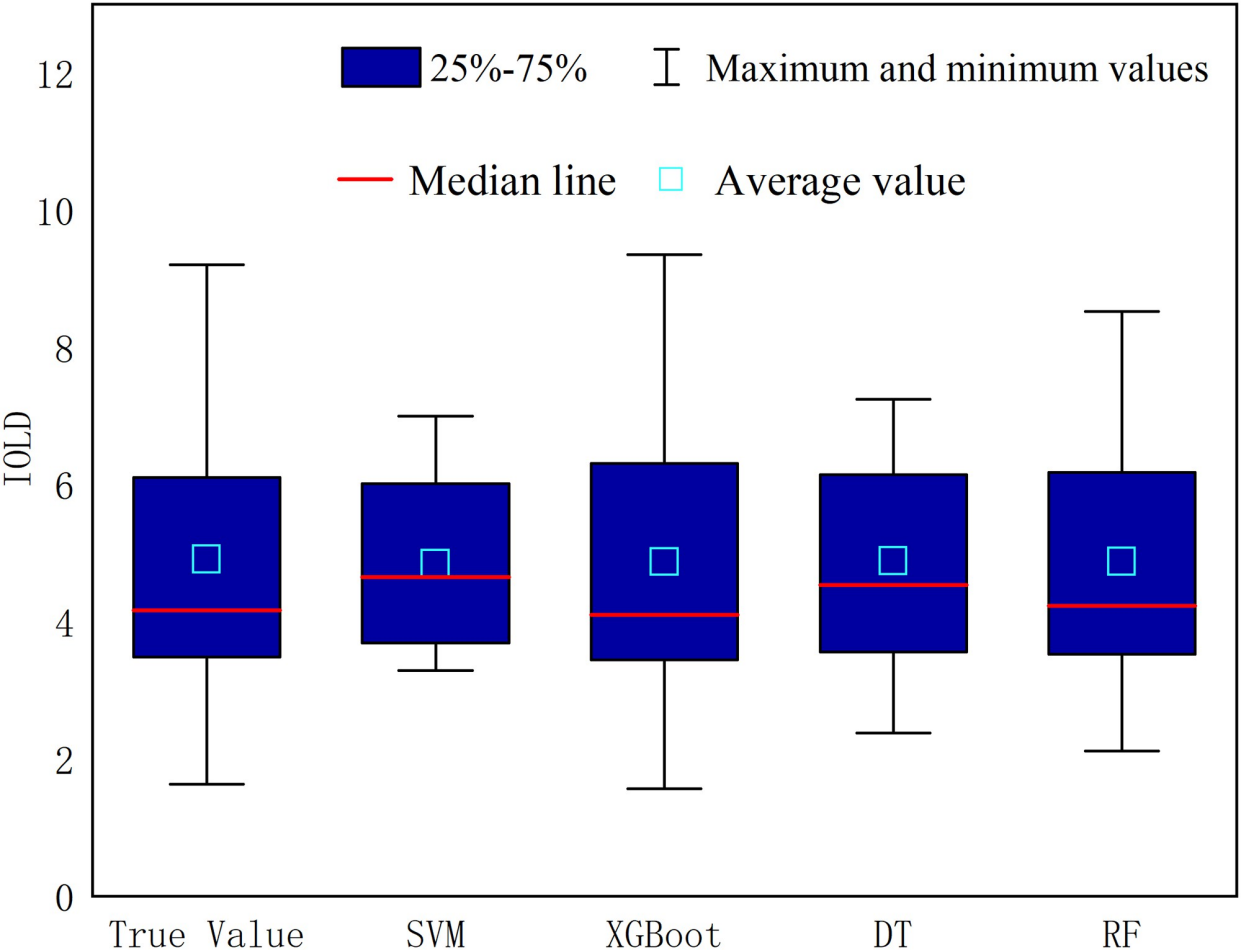
Comparison between the predicted real value and predicted value of the model.

Further, after learning the attribute characteristics of the model for land development intensity and influencing factors, the simulated values of the four models were evaluated using the test set. The simulated values of land development intensity for each model were plotted against the valid values in a box line plot (Fig 4). It can be seen that the mean values of the estimates of the four models do not differ significantly from the valid values, and the median of the actual values is almost co-linear with the XGBOOST and Random Forest models, which shows consistency with the training learning change curves of the two models. However, in terms of maximum and minimum values, the actual values are most similar to the predicted values of the XGBOOST model.

In the process of continuous optimization of the model algorithm, select the test parameter comparison when the four models predict the performance. The XGBOOST algorithm’s R^2^ (95.66%) is the largest, while MSE (0.1622) is the smallest, which shows that its accuracy is better than the other three models. Based on the above comparison, the XGBOOST algorithm was finally selected to construct the land development intensity prediction model.

## 4. Model accuracy validation

### 4.1. XGBOOST algorithm’s hyperparameter search optimization validation

**Fig 5:**
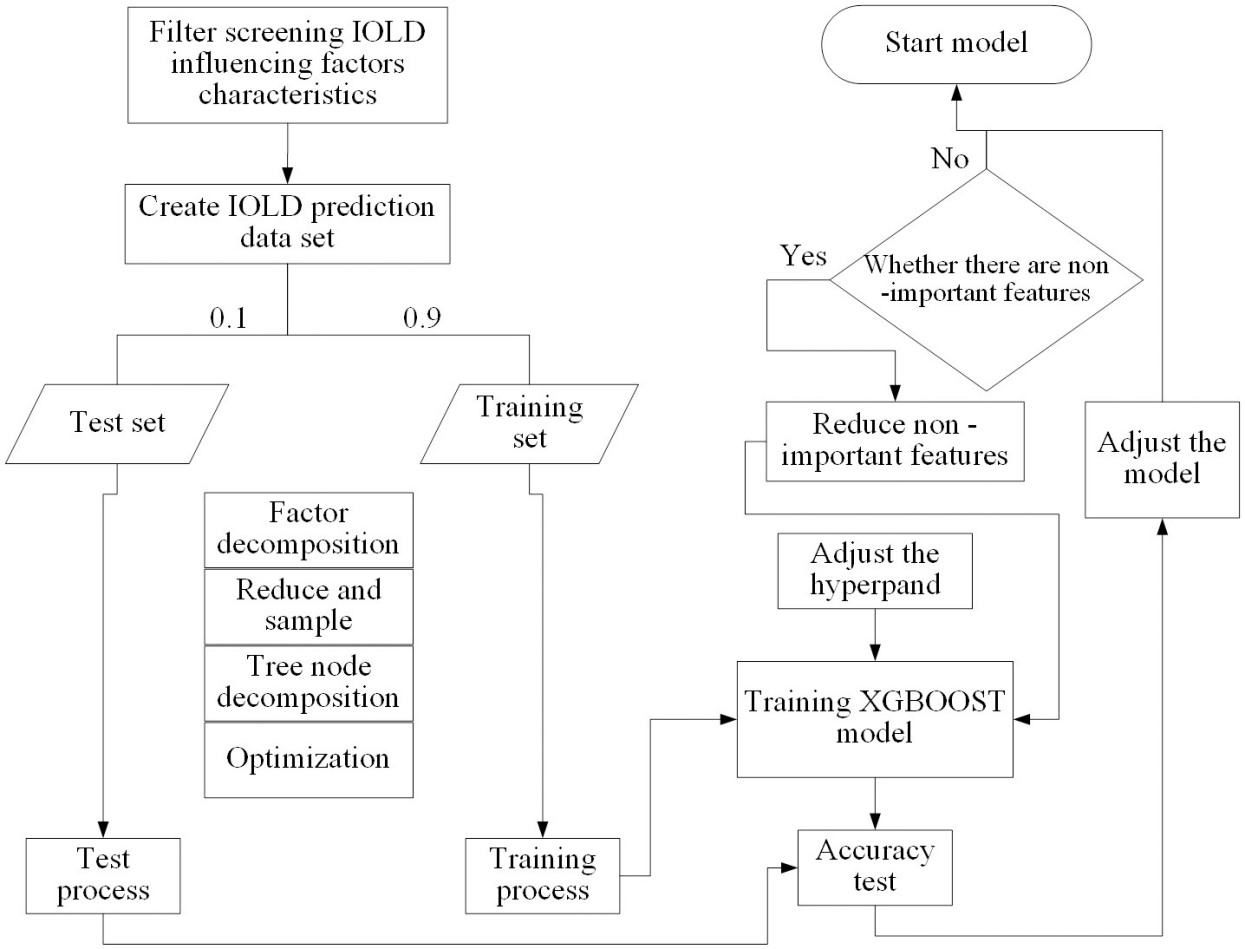
Modeling flowchart of XGBOOST based on land development intensity.

The principle and modeling process of the XGBOOST algorithm (Fig 5) shows that hyperparameters such as max-depth, learning rate, and n-estimators are critical to the training performance of the XGBOOST algorithm. When the initial values of hyperparameters are set to train the XGBOOST algorithm, the corresponding models are generated based on the pre-processed training set. Nevertheless, how to prove that the current hyperparameters are the best combination? It will be verified in the following section.

In the training model learning process, the k-fold cross-validation method is used to improve the training performance by randomly dividing the training set of land development intensity into k different subsets. An array of k evaluation scores is obtained based on training and evaluating the established XGBOOST model k times, selecting one fold at a time for evaluation and training on the other (k-1) folds. At the same time, new learners are added to the fold, and during the iteration, the new learners correct the previously predicted values and produce a new deal to optimize the model further.

The XGBOOST algorithm will narrow the search and improve the fine-tuning efficiency to further determine the best parameter for the model. A coarse search for an extensive range of hyperparameters with a loose common difference is performed based on the initial values. We arrange the parameter combination of the model to identify the vague field of different super parameters. Finally, find the combination of the super-reuse when the predictive model realizes the maximum accuracy.

**Fig 6:**
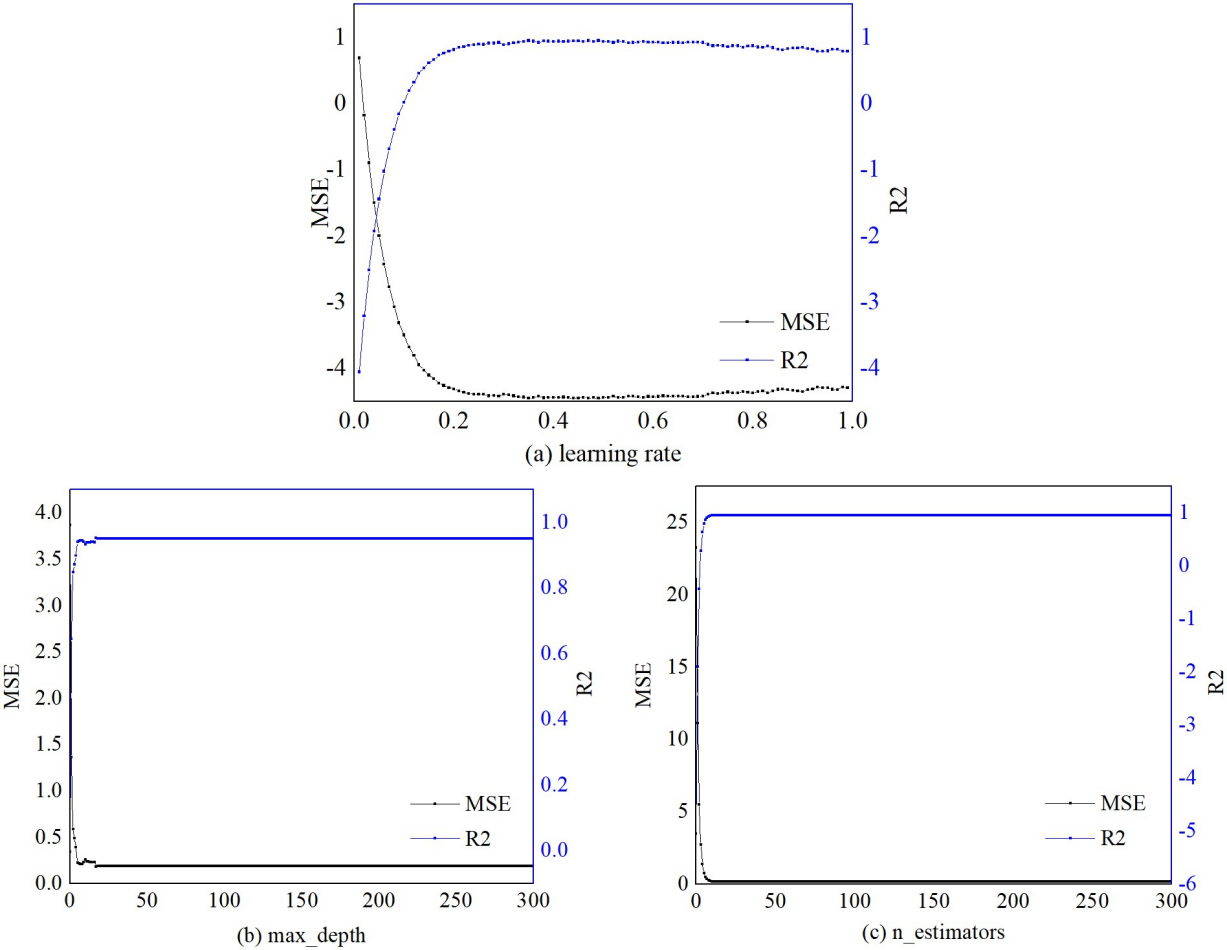
XGBOOST model parameters in the process of training the learning curve.

In the process of debugging the parameters, each hyperparameter forms its learning curve, and the mean square error and bias are used to assess the accuracy of the training land development intensity prediction process. From the changes in the learning curves of parameters such as learning rate, max-depth, and n-estimators in Fig 6, it is clear that the prediction model increases the value of R^2^ as the training set increases in the process of continuous learning. The final training learning curve approaches 1, while the MSE converges rapidly in continuous iterations and remains constant after reaching the minimum value. From the combination of hyperparameters of the XGBOOST model (Table 4), when their values are 0.47, 19, and 10, respectively, the mean square error is the smallest, and the goodness of fit is the highest, and the model simulation can appear as the best prediction solution.

**Table 4:** XGBOOST super parameter combination of the model.

Parameter	learning_rate	max_depth	n_estimatiors
Value	0.47	19	10

### 4.2. Model accuracy evaluation

The machine learning algorithm is randomly divided by the three labels of the "region," "province," and "year" of the experimental data set. The description of the test set is shown in Table 5. It can be found that the test samples are not sampled equally, but the test samples are randomly divided by analyzing the attribute characteristics of each model, which are distributed in all seven geographical divisions. However, it shows randomness in terms of provinces and years, and random sampling can avoid the interference of human factors on the training effect and can more effectively test the land development intensity prediction model’s generalization ability.

**Table 5:** XGBOOST model test set verification results description.

Region	Provinces involved	Years involved	Average true value	Average predicted value	Difference value	MSE	R^2^
Northeast Region	Heilongjiang, Jilin, and Liaoning	2006a, 2007a, 2011a, 2012a, 2014a, 2016a, 2020a	3.14	3.29	0.14	0.16	95.66%
North China	Hebei, Inner Mongolia, Shanxi, Tianjin	2002a, 2006a, 2008a, 2009a, 2013a	4.4	4.14	-0.27		
East China	Anhui, Fujian, Jiangsu, Jiangxi, Shandong	2005a, 2006a, 2008a, 2009a, 2014a, 2015a, 2017a, 2018a, 2019a	5.86	5.63	-0.24		
South China	Guangdong, Guangxi, Hainan	2002a, 2005a, 2006a, 2010a, 2012a, 2018a, 2019a	5.07	5.29	0.22		
Central China	Henan, Hubei, Hunan	2003a, 2006a, 2007a, 2008a, 2009a, 2015a, 2018a, 2019a, 2020a	6.01	5.79	-0.21		
Northwest China	Gansu, Ningxia, Qinghai, Shaanxi, Xinjiang	2003a, 2004a, 2010a, 2012a, 2013a, 2014a, 2015a, 2017a, 2018a	3.76	3.81	0.06		
Southwest Region	Sichuan, Yunnan, Chongqing	2002a, 2004a, 2014a, 2015a, 2018a, 2019a	5.53	5.43	-0.1		

**Fig 7:**
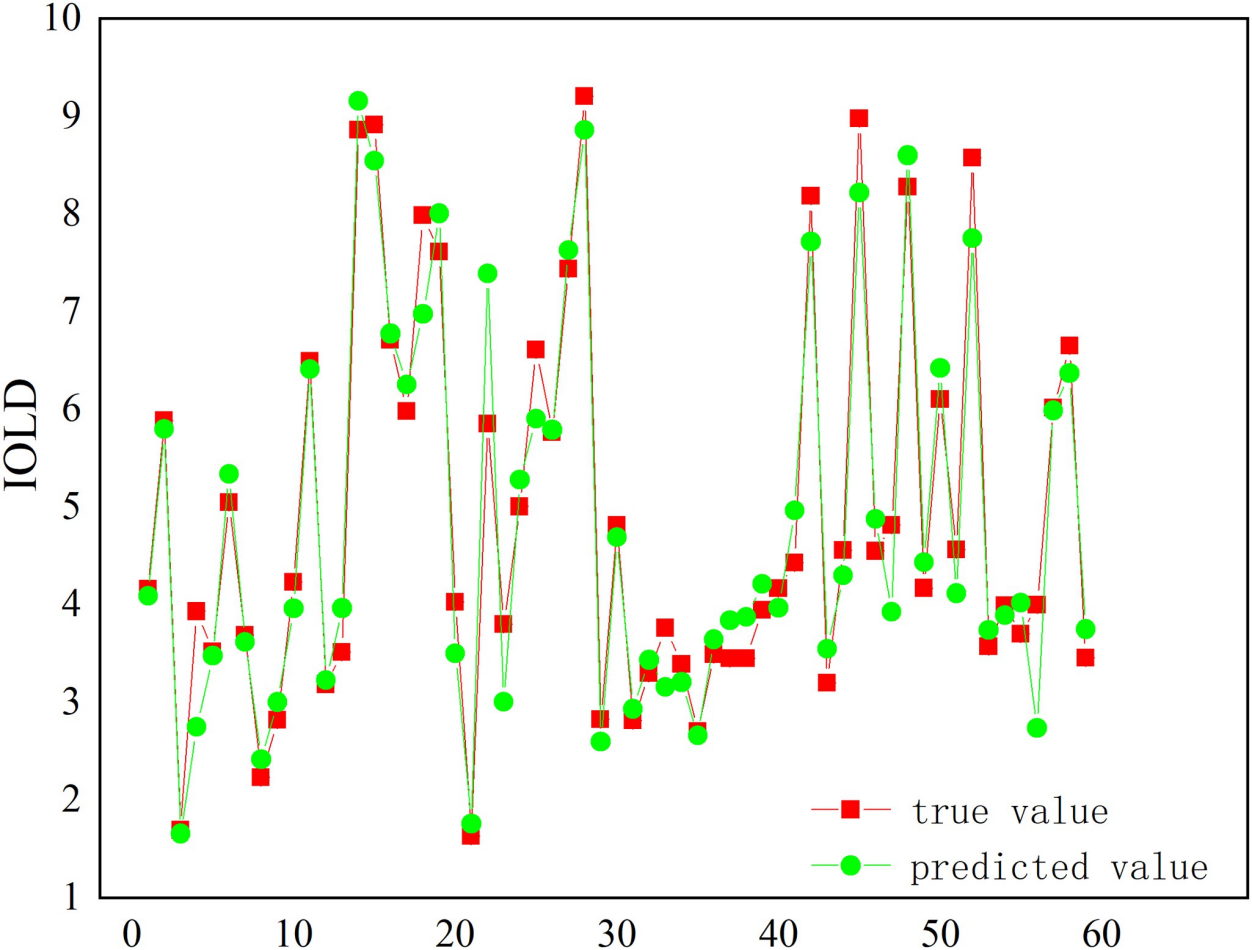
The fitting map of land development intensity test based on the XGBOOST model.

The test set data is deployed to the trained XGBOOST prediction model to derive the predicted values of the target. From the difference between the actual mean value and the predicted mean value of the model in each region, the overall value is close to the actual value. The most significant difference is -0.27 in North China, while the minor difference is 0.06 in Northwest China, which is related to the geographical distribution of land development intensity, as the level of land development intensity in North China is at the top of the seven geographical divisions in China, however, the level difference within the region is also more significant. In contrast, the level of land development intensity in Northwest China is more backward and relatively evenly distributed, which makes the predicted differences divergent. The linear fits of the predicted and actual values are then plotted in Fig 7, and the MSE and R^2^ values are calculated to assess the goodness of fit, and it can be learned that the predicted and actual values have a good fit with R^2^ = 95.66% and MSE = 0.16. Accordingly, the prediction results of the land development intensity prediction model are highly accurate and do not show any under-fitting or over-fitting.

## 5. Conclusion and discussion

### 5.1. Conclusion

This paper simulates the prediction of land development intensity in China based on four machine learning models (XGBOOST, random forest model, support vector machine, and decision tree) and finally selects the XGBOOST algorithm with the highest prediction accuracy for building the prediction model by comparing the experimental results, and carries out the verification of hyperparameter combination and prediction results based on the XGBOOST model.

Comparing the regression fits of the training and test datasets of each model, XGBOOST has the best prediction performance with a high coefficient (95.66%) and low mean square error (0.1622) evaluation scores, but the overall prediction of the random forest model (R^2^ = 94.72%, MSE = 0.1654) is close to the accuracy of the XGBOOST algorithm and much greater than the remaining two models. However, in terms of the learning curves’ stability, using XGBOOST for land development intensity prediction can produce better results.

The combination of hyperparameters of the XGBOOST model plays a decisive role in the model’s prediction accuracy. Hyperparameter debugging ranges from coarse search over an extensive range to fine-tuning of parameters in small intervals, combined with the evaluation of the learning curve variation of the training set and the error and bias. The optimal combination of hyperparameters (max_depth: 19, learning_rate: 0.47, n_estimatiors: 10) for the XGBOOST algorithm-based land development intensity prediction model was determined using the test value error further to validate the scientificity of the model parameter determination.

### 5.2. Discussion

The XGBOOST algorithm has proven its effectiveness in numerous machine learning and data mining challenges for prediction and classification problems and was rated as the top solution algorithm in a machine learning competition held on the Kaggle website [37], with the main advantages being the minimum requirement for attribute normalization, intelligent handling of missing values, and providing solutions that avoid overfitting [38,39]. The land development intensity prediction model constructed based on the XGBOOST algorithm in this paper shows better effect advantages in simulation accuracy and operation speed and provides a more accurate and intelligent prediction method for the dynamic Simulation of land development and utilization.

It should be noted that although machine learning algorithms have some advantages over traditional statistical methods in improving the accuracy of land development intensity prediction, these algorithms mostly make numerical predictions based on the interaction of data itself. Therefore, machine learning methods should be used cautiously to deeply reveal geographic factors’ driving mechanism or causal relationship on land development intensity. In future land development intensity simulation studies, traditional statistical methods of land development intensity can be combined with machine learning algorithms, with the former used to explain the formation mechanism of the spatial distribution of land and select predictor variables and the latter better performing the task of land development intensity prediction. It may be able to improve the accuracy of land development intensity simulation and enhance the interpretability of the model at the same time. At the same time, further exploring the spatial differentiation and formation mechanism of land development intensity and more reasonably selecting the influencing factors of land development intensity are also important research directions to improve the accuracy of land development intensity distribution prediction in the future. In addition, the prediction object of this paper is the land development intensity of a large-scale provincial unit. The subsequent research will consider collecting samples from municipal scale, county scale, urban clusters, economic zones, and other research units for training to improve the data and universality of the samples, further test and improve the simulation performance of the XGBoost prediction land development intensity model. And form a continuous spatial distribution atlas of regional land development intensity to support the construction of a dynamic monitoring system of urban land development in the era of big data.
